# Evaluative audiometry after cochlear implant provision

**DOI:** 10.1007/s00106-023-01317-7

**Published:** 2023-10-09

**Authors:** Oliver C. Dziemba, Stephan Merz, Thomas Hocke

**Affiliations:** 1 Klinik und Poliklinik für Hals‑, Nasen‑, Ohrenkrankheiten, Kopf- und Halschirurgie, Universitätsmedizin Greifswald, Ferdinand-Sauerbruch-Straße, 17475 Greifswald, Germany; 2 Merz Medizintechnik, Reutlingen, Germany; 3 Cochlear Deutschland GmbH & Co. KG, Hannover, Germany

**Keywords:** Prostheses and implants, Patient-specific modeling, Loudness scaling, Speech audiometry, Computer simulation

## Abstract

**Background:**

One of the main treatment goals in cochlear implant (CI) patients is to improve speech perception. One of the target parameters is speech intelligibility in quiet. However, treatment results show a high variability, which has not been sufficiently explained so far. The aim of this noninterventional retrospective study was to elucidate this variability using a selected population of patients in whom etiology was not expected to have a negative impact on postoperative speech intelligibility.

**Materials and methods:**

Audiometric findings of the CI follow-up of 28 adult patients after 6 months of CI experience were evaluated. These were related to the preoperative audiometric examination and evaluated with respect to a recently published predictive model for the postoperative monosyllabic score.

**Results:**

Inclusion of postoperative categorical loudness scaling and hearing loss for Freiburg numbers in the model explained 55% of the variability in fitting outcomes with respect to monosyllabic word recognition.

**Conclusion:**

The results of this study suggest that much of the cause of variability in fitting outcomes can be captured by systematic postoperative audiometric checks. Immediate conclusions for CI system fitting adjustments may be drawn from these results. However, the extent to which these are accepted by individual patients and thus lead to an improvement in outcome must be subject of further studies, preferably prospective.

## Introduction

Treatment with a cochlear implant (CI) represents a therapy option for patients who suffer from a higher degree of sensorineural hearing loss and for whom one expects a better outcome with the CI than with other therapies [5]. For the invasive type of CI therapy, it is therefore of great importance to estimate the expected postoperative speech intelligibility as accurately as possible already at the time of the indication.

When CI provision first began, the potential of preoperative differential diagnosis was limited, as the indication was usually only given in cases of bilateral, complete functional deafness [8]. Although patients with functional residual hearing have increasingly been provided with a CI in recent decades, it is hardly surprising that prediction studies with large case numbers [1, 17, 21] have identified anamnestic, etiologic, and surgical factors as the strongest influencing variables: Residual hearing, if present, played a relatively minor role in these studies. Blamey et al. [1] identified five factors that have a significant influence on the expected speech intelligibility:

- The duration of the deafness
- The age of onset of profound hearing loss
- The age at the time of CI fitting
- The etiology
- The hearing experience with CI

Developments in technology, audiology, surgery, and rehabilitation [4, 7, 12, 15, 16, 19, 22–26, 29] have led to better outcomes in the past decade. As a result, hearing aid users with still usable speech intelligibility have been indicated for CI, and their outcome has significantly exceeded that of the hearing aid fitting. Thus, the realistic prediction of the foreseeable speech intelligibility plays an important role in preoperative counseling of these patients. With considerable residual hearing—i.e., in Germany up to 60% monaural monosyllabic word recognition score (WRS) at 65 dB_SPL_ with the best-fitting hearing aid, WRS_65_(HA)—reliable predictions based on preoperative findings are becoming increasingly important for the indication, counseling, and quality control [20, 28].

Hoppe et al. [18] showed that an individual prediction of the achievable speech intelligibility following CI intervention is possible. In their study, a generalized linear model (GLM) was applied. This GLM is based on the preoperatively measured maximum word recognition score (WRS_max_), WRS_65_(HA), and age at implantation. Hoppe et al. [18] also pointed out the large variability in the data. However, despite this variability, clinically relevant statements applicable to individual cases can be derived [18, 20]. For example, three quarters of the CI patients that were studied achieved or exceeded the prediction within a window of −12 percentage points (pp; [18]). A more extensive and detailed analysis of the possible causes for the variability found in the data was not conducted. For this purpose, the combination of the GLM with the results of a study by Blamey et al. [1] is useful. They were able to show that certain etiologies might have a negative impact on hearing outcome with CI. If patients with the negative-acting etiologies described in this study were excluded from the model, the part of the variability that could be explained by extrinsic factors would inevitably increase. Thus, if patients in this population perform below the prediction, specific aspects of CI fitting, rehabilitation, and process control will certainly have a stronger influence: The differences to the prediction will be largely explained by the data from the clinical–audiological evaluation of the processor settings.

Thus, the aim of the present work was to investigate the relationship between postoperative word recognition and its preoperative prognosis. For the analysis of this variability, further clinical–audiological obligatory parameters were used, such as loudness scaling and determination of the hearing loss for multisyllabic Freiburg numbers. Following the above reasoning, patients with an etiology whose influence suggests a below-average fitting outcome were excluded. As a consequence, the influence of possible extrinsic factors in the study population becomes more apparent.

## Material and methods

### Inclusion criteria and study participants

**Fig. 1:**
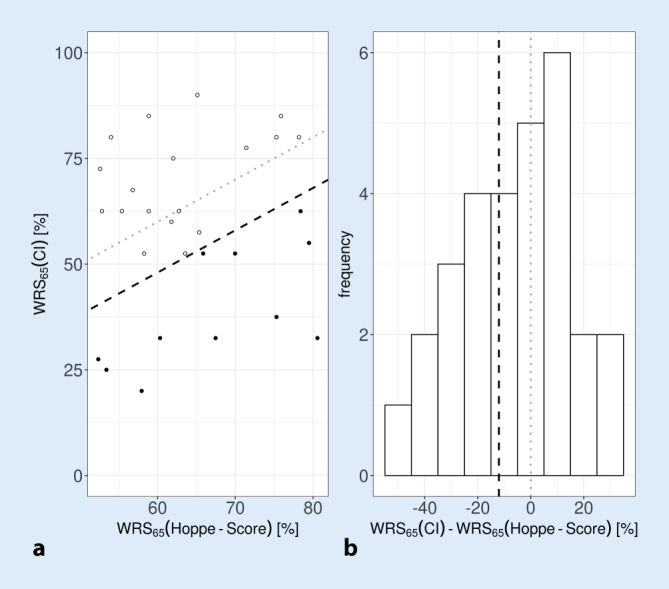
Scatter plot (**a**) and histogram (**b**) according to Hoppe et al. [18]. *Dotted line* shows the bisecting line. *Dashed line* shows the 25% outcome quartile according to Hoppe et al. [18]. In the scatter plot, group 1 is shown with *open* *circles*, group 2 with *filled circles*. *WRS* word recognition score

The inclusion criteria for retrospective data analysis were defined as follows:

- Age of at least 18 years at the time of CI provision
- Etiology with a mean percentile rank of ≥ 0% according to Blamey et al. (Figure 6; [1])
  - Sudden idiopathic, genetic, Meniere’s disease, otosclerosis, unknown, acoustic trauma, miscellaneous
- Preoperative data available:
  - Unaided WRS_max_
  - WRS_65_(HA)
- audiometric findings available at 6 months after initial fitting
  - Hearing loss for number words (German: HVZ): The HVZ is the difference between the individual measured speech recognition thresholds (SRT) in quiet for Freiburg multisyllabic numbers and the normal hearing reference
  - Freiburg monosyllabic score in quiet in free field at 50/65/80 dB_SPL_
  - Monaural speech reception threshold L_50_ in noise with the Oldenburg Sentence Test (OLSA)
  - Loudness scaling in the frequencies 250/500/1000/2000/4000 Hz
- Signed patient consent for anonymous data processing of clinical routine data

Explicitly excluded were cases with the etiologies of ototoxicity, labyrinthitis, chronic otitis media, meningitis, temporal bone fracture, Schwannomas, auditory synaptopathy, and neuropathy, whose outcomes remain comparatively below average according to Blamey et al. [1].

This study included 29 CI provisions in 28 patients (12 male, 16 female). In the case of a bilateral-sequential CI provision, both sides could be included in the evaluation. The cases were grouped into 13 left-sided and 16 right-sided fittings. The mean age at the time of surgery was 59.3 years (min. 30 years, max. 81 years). There was no selection according to CI manufacturer. The numbers per CI manufacturer are shown in Table 1.

**Table 1:** Number of included cochlear implant supplies per manufacturer

Manufacturer	Quantity
Advanced Bionics	3
Cochlear	19
MED-EL	7

### Preoperative prediction of the outcome for quiet

To estimate the individual word recognition score with CI in quiet at 65 dB_SPL_—WRS_65_(CI)—after a period of approximately 6 months from the preoperative audiometric data, the calculation was performed according to Eq. 1 [18]. This predictive value will be referred to as the “Hoppe score” in the following. The necessary coefficients (β values) are shown in Table 2.

1 $$\begin{aligned}\kern-4mm&\mathrm{WRS}_{65}\left(\mathrm{CI}\right)\left[\mathrm{{\%}}\right]=\\\kern-4mm&\frac{100}{1+e^{-\left(\beta _{0}+\beta _{1}\cdot \mathrm{WRS}_{\max }+\beta _{2}\cdot \mathrm{Age}+\beta _{3}\cdot \mathrm{WRS}_{65}(\mathrm{HA})\right)}}\end{aligned}$$

**Table 2:** Coefficients for the calculation of the Hoppe score according to Eq. 1 [18]

Coefficient	Value	[β]
β_0_	0.84	–
β_1_	0.012	1/%
β_2_	−0.0094	1/year
β_3_	0.0059	1/%

Positive β values mean a positive influence of the corresponding variables on the prognosis and vice versa

### Audiometric measurements with CI

All audiometric measurements were performed in rooms that fully met the requirements of the DIN EN ISO 8253. The MA55 audiometer (MAICO Diagnostics GmbH, Berlin, Germany) was installed at all measurement sites. The headphones used were DT48 (beyerdynamic GmbH & Co. KG, Heilbronn, Germany) or PD-95 (Holmberg GmbH & Co. KG, Berlin, Germany). Due to the regular technical checks (MTK) and receiver-dependent free-field correction, deviations in the results between the headphones used can be neglected.

For preoperative speech audiometry with hearing aid in the free sound field, the loudspeaker 8020D (GENELEC®, Iisalmi, Finland) was used. All postoperative audiometric measurements with CI in the free sound field were performed using the LAB-251 loudspeaker (Westra Elektroakustik GmbH, Wertingen, Germany).

#### Speech audiometry in quiet

Speech intelligibility in quiet was measured using the Freiburg Speech Test [14], which consists of 10 lists of multisyllabic numbers and 20 lists of monosyllabic words. The preoperative measurement of the WRS_max_ was performed unilaterally via headphones. The WRS_max_ is obtained as a maximum score from the measured discrimination function of the Freiburg monosyllabic test. The WRS_65_(HA) was measured with the Freiburg monosyllabic test in free sound field with a distance to the loudspeaker of 1 m and a frontal sound presentation. Possibly occurring crosstalk was suppressed by the generally used procedure via headphones.

As part of the postoperative evaluation of speech audiometry in quiet, the HVZ and monosyllabic scores for speech sound levels 50/65/80 dB_SPL_ were measured. This procedure is a clinical standard at the first author’s institution and has been described several times [9, 11].

#### Loudness scaling

To represent the suprathreshold dynamic with CI, a loudness scaling according to DIN ISO 16832 [6] can be measured. This loudness scaling was adaptively measured with the Oldenburg measurement program OMA (HörTech gGmbH, Oldenburg, Germany) version 1.5.5.0 [2]. As described by Dziemba et al. [11], curves of equal loudness of auditory perception for the measured frequency range can be obtained from the loudness scaling by regression. An 11-level scale—*very quiet* (5 CU, categorial unit), quiet (15 CU), *medium* (25 CU), *loud* (35 CU), *very loud* (45 CU), and *too loud* (50 CU)—was used to represent loudness categories.

In order to separate the determination of the frequency-specific hearing threshold with CI from the slope of the level-loudness function, the hearing threshold determination from the auditory field scaling according to Rader et al. [25] was applied.

The extraction of the raw data of all measurements was performed with a proprietary software module from Merz Medizintechnik GmbH (Reutlingen, Germany).

#### Speech audiometry in noise

The OLSA is a matrix test based on the model of Hagermann [13], which was adapted for the German language, optimized and evaluated for measurements in noise in a reference situation [30–32]. The OLSA can be used to adaptively measure SRT in noise. The adaptive measurement of a 50% speech recognition threshold (L_50_) in noise is performed by varying the presentation level of one signal component (speech or noise), while the other signal (noise or speech) remains fixed in the presentation level [3]. According to our investigations [9] for monaural speech audiometry in noise with the OLSA, the speech signal was kept fixed at 65 dB_SPL_. The difference between the speech sound level and the level in the L_50_ results in the maximum acceptable noise level (ANL) at the SRT in noise.

The postoperative, monaural measurement of ANL with CI was performed with the Oldenburg measurement program OMA (HörTech gGmbH, Oldenburg, Germany) version 1.5.5.0. The methodology for all measurements was followed according to the in-house standard in analogy to Dziemba et al. [9].

## Results

### Illustration according to Hoppe et al. [18]

In Fig. 1, the WRS_65_(CI) scores at 6 months postoperatively are shown versus the prognosis in the scatter plot (a) and as a histogram (b), according to Hoppe et al. [18]. The dashed lines show the bisecting line (gray) and the first quartile at −12 pp for the WRS_65_(CI) from Hoppe et al. ([18]; black) in the scatter plot and in the histogram.

We used the first quartile of the Hoppe score from the original paper [18] as a cut-off criterion for outcome. Thus, the population is divided into 18 CI fittings for which the predicted fitting outcome can be considered achieved (group 1). In 11 CI fittings, the predicted WRS was missed by more than 12 pp (group 2). It is remarkable that according to this dynamic and individual definition, four cases with open speech understanding, WRS_65_(CI) > 50%, have to be assigned to group 2, i.e., as cases with therapy goals not yet achieved.

### Loudness scaling

**Fig. 2:**
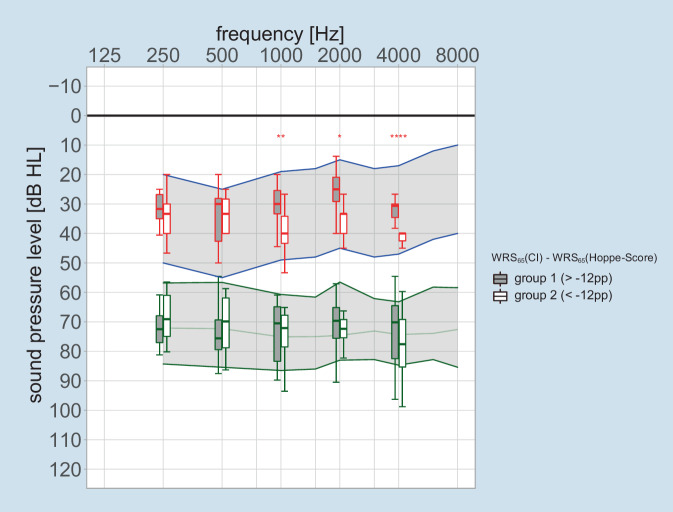
*Blue-bordered* *gray-shaded* area shows the speech level field for representative normal-loud German speech (L_eq_ = 65 dB_SPL_) according to Steffens [27]. *Green-bordered* *gray-shaded* area shows the reference (mean and 95%confidence interval) of the loudness scaling for a categorical loudness of 25 CU according to DIN ISO 16832 [6]. The measured values are presented in *grouped box plots*. *Boxes* show the median and the quartiles. *Whiskers* show the maximum measured value within 1.5 times the interquartile range. *Asterisks* indicate significance level in Student’s *t *test comparing the two groups (*p < 0.05; **p < 0.01; ***p < 0.001; ****p < 0.00001). *Red* grouped measured values of hearing threshold from the loudness scaling according to Rader et al. [25]. *Green* grouped measured values of categorical loudness of 25 CU. *CI* cochlear implant, *WRS* word recognition score

Figure 2 shows the hearing threshold with CI according to Rader et al. [25] and the levels of equally loud auditory perception with CI for the loudness category “medium” (25 CU) for the frequencies 250/500/1000/2000/4000 Hz grouped by group 1 and group 2 after 6 months as boxplots. As a target value postulated by the authors for the levels of medium-loud perception with CI, the reference values of the loudness scaling of normal hearing persons from DIN ISO 16832 [6] are shown as a green-bordered and gray-shaded area.

To illustrate the effects of different, near-threshold auditory perceptions on speech intelligibility, the corresponding area for representative normal-loud German speech (L_eq_ = 65 dB_SPL_) according to Steffens [27] is shown as a blue-bordered area with a gray background.

While there were no significant group differences for the levels of medium-loud perception, significant to highly significant differences between the two groups were found at the hearing threshold. These differences of the near-threshold perception are significant in the frequency range from 1 to 4 kHz only.

### Speech intelligibility in quiet

**Fig. 3:**
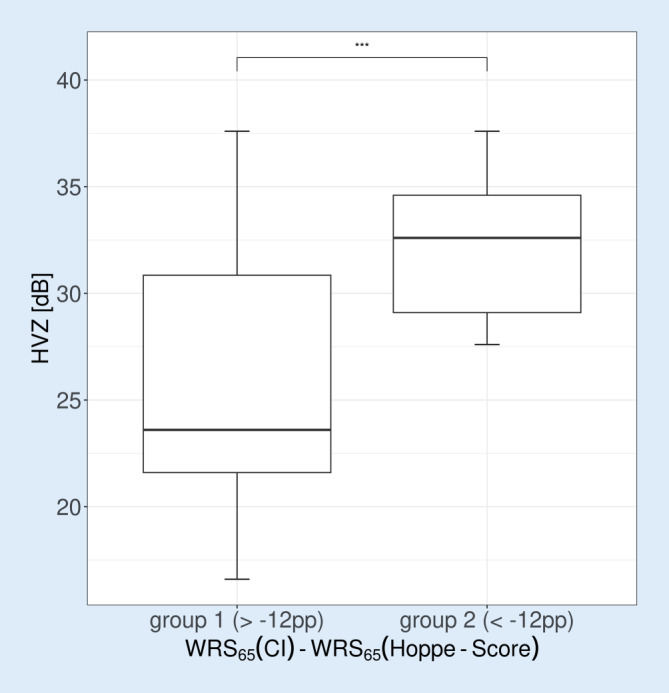
Grouped box plots of hearing loss for numbers (*HVZ*) with cochlear implant (*CI*). *Boxes* show the median and quartiles. *Whiskers* show the maximum measured value within 1.5 times the interquartile range. *Asterisks* indicate significance level in Student’s *t* test (*p < 0.05; **p < 0.01; ***p < 0.001; ****p < 0.00001). *WRS* word recognition score

The grouped representation of the HVZ with CI after 6 months is shown in Fig. 3. There was a highly significant difference in the HVZ between groups and a larger variability of the results in group 1.

**Fig. 4:**
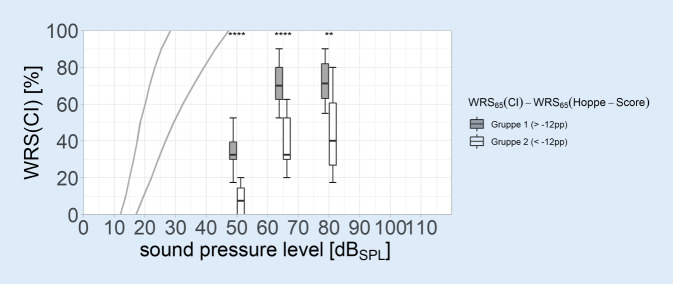
Grouped boxplots of Freiburg monosyllabic scores in quiet with cochlear implant (*CI*) at 50/65/80 dB_SPL_. *Boxes* show the median and the quartiles. *Whiskers* show the maximum measured value within 1.5 times the interquartile range. *Asterisks* indicate significance level in Student’s *t *test (*p < 0.05; **p < 0.01; ***p < 0.001; ****p < 0.00001). *WRS* word recognition score

The monosyllabic word recognition scores with CI after 6 months are shown in Fig. 4 for each group. Trivially enough by group assignment, the group difference for WRS_65_(CI) was significant. This difference between group 1 and group 2 is also shown for the flanking speech sound levels, but at different significance levels.

### Speech intelligibility in noise

**Fig. 5:**
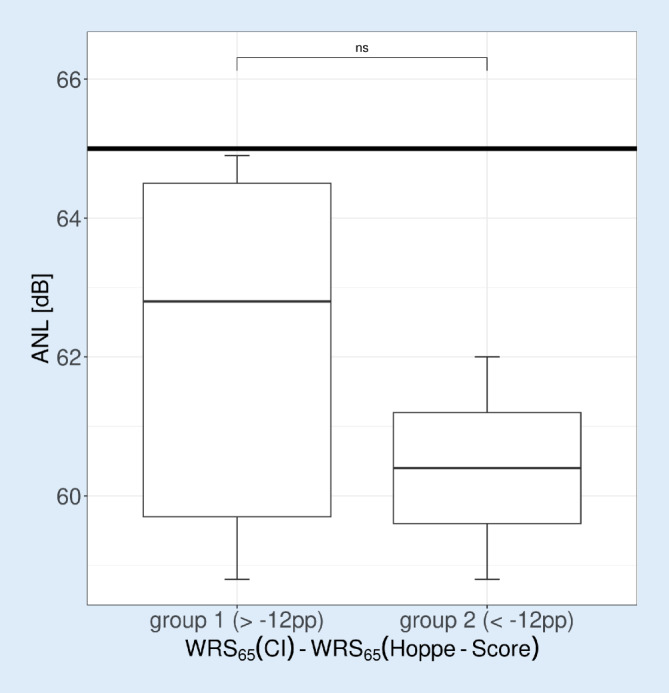
Grouped boxplots of the acceptable noise level (*ANL*) calculated from the speech recognition threshold L_50_ of the Oldenburg Sentence Test at a fixed speech level of 65 dB_SPL_. *Boxes* show the median and quartiles. *Whiskers* show the maximum measured value within 1.5 times the interquartile range. *Horizontal line* at 65 dB marks the fixed speech level and thus an L_50_ of 0 dB. No significant difference was found. *CI* cochlear implant, *WRS* word recognition score

The data shown in Fig. 5 complete the results of postoperative audiometry with CI described in the previous figures.

In Fig. 5, the ANL according to Dziemba et al. [9] is shown for the two groups as boxplots. There are no significant differences between the two groups for this measurement.

### Extension of the GLM

To explain the deviations in the measured 6‑month values from the predicted WRS (Eq. 1), the existing GLM was extended by the following postoperatively measured variables: the percentage hearing loss (pHV) as calculated from the loudness scaling and the HVZ. The strongly suprathreshold measured values from the loudness scaling for 25 CU were not included in the model (*p* = 0.52). Thus, Eq. 2 results in:

2 $$\mathrm{WRS}_{65}\left(\mathrm{CI}\right)\left[\mathrm{{\%}}\right]=\frac{100}{1+e^{-\left(\beta _{0}+\beta _{1}\cdot \mathrm{WRS}_{\max}+\beta _{2}\cdot \text{Age}+\beta _{3}\cdot \mathrm{WRS}_{65}(\mathrm{HA}){+\gamma _{0}}+\gamma _{1}\cdot pHV+\gamma _{2}\cdot HVZ\right)}}$$

with the values shown in Table 3 listed factors γ.

**Table 3:** Model parameters

		Value	SD	*t* Statistics	*p*
*Constant*	γ_0_	2.98	±0.47	6.33	2.47e^−10^
*pHV*_*Rader*_	γ_1_	−0.031	±0.0096	−3.27	0.0011
*HVZ*	γ_2_	−0.070	±0.014	−5.06	4.26e^−07^

*HVZ* hearing loss for number words, *pHV* percentage hearing loss *SD* standard deviation

**Fig. 6:**
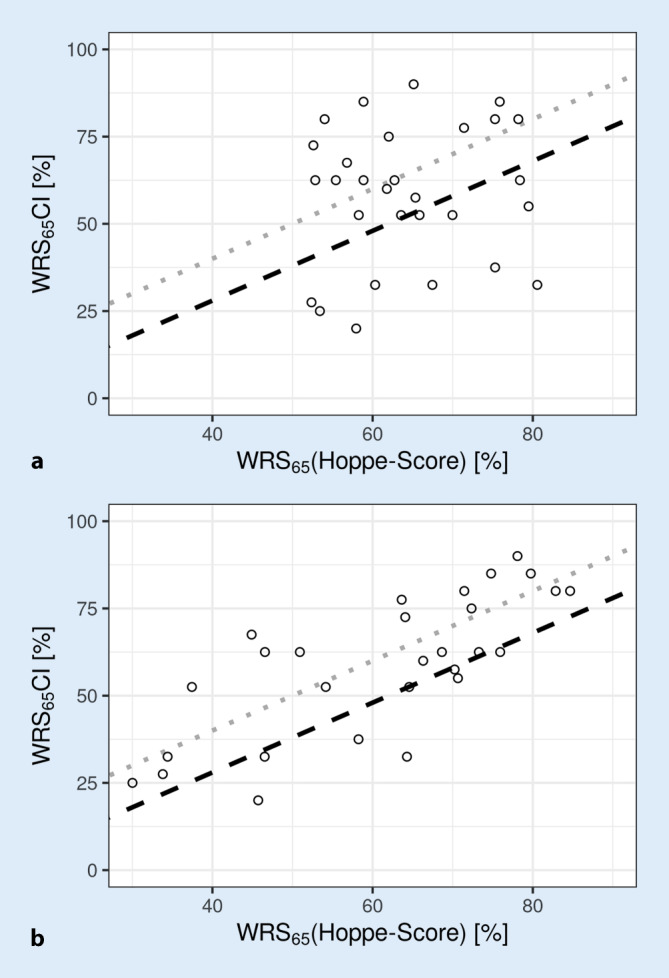
WRS_65_(CI), 6 months postoperatively, plotted in relation to predicted WRS according to Eq. 1 (**a**) and Eq. 2 (**b**). For the *upper part*, there is no correlation between prognosis and achieved speech intelligibility (*R* = 0.098, *p* = 0.61). For the *lower part*, taking into account the percentage hearing loss and the hearing loss for numbers, there is a significant correlation (*R* = 0.74, *p* = 4∙10^−6^). *CI* cochlear implant, *WRS* word recognition score

Figure 6 shows the WRS_65_(CI), 6 months postoperatively, each above the predicted speech scores according to Eq. 1 (Fig. 6a) and Eq. 2 (Fig. 6b). Whereas the data in Fig. 6a do not correlate (*R*_Spearman_ = 0.098, *p* = 0.61), adding the fitting-related variables listed above resulted in a significant relationship (*R*_Spearman_ = 0.74, *p* = 4∙10^−6^). The large variability shown in Fig. 6a can now be explained by 55% of the fitting of the CI system that can be influenced in principle.

## Discussion

In the present study, a model for predicting speech intelligibility after cochlear implantation was applied to a selected population of CI users. Patients with an etiology that could potentially negatively influence outcome (according to Blamey et al. [1]) were excluded so as to investigate possible fitting-related causes for deviations from predicted speech intelligibility.

We found that the variability in outcome is to a considerable extent caused by potentially optimizable settings of the CI systems, especially in the range of loudness close to the threshold.

No correlation was found between predicted and measured word recognition scores after 6 months (Fig. 1a). The important finding here is that by adding simple data from the audiometric evaluation, 55% of the variability in outcome can be explained. In this way, the GLM from Eq. 1 was transformed from a predictive model to an explanatory model according to Eq. 2. However, the significant influence of the postoperatively measured variables, in this case HVZ and curves of the same loudness category, or the nonsignificant influence of other variables on the achieved monosyllabic scores harbors a potential fallacy. In a model that “must” explain post hoc variability in results, there are two possible interpretations. As soon as a factor, such as a fitting parameter, occurs almost identically in the population under study, the same factor cannot lead to a significant test result for the explanation of the variability. This property of the analysis by means of GLM is not equivalent to a loss of significance of this very factor. Therefore, only a non-optimal setting of a certain factor would cause the corresponding variability in the results and thus be identified by the extended GLM as a significant influencing variable. In this study, for example, the adjusted dynamic range of the CI system had no significant influence on the word recognition according to the extended GLM. Apparently, the small deviations in the medium-loud category (25 CU) do not explain the differences found in speech intelligibility, either due to a sufficiently good setting of the systems in this regard or due to insufficient case numbers. Thus, the near-threshold results of the loudness scaling in Fig. 2 for group 2 over the entire frequency range show that audibility is only achieved at levels that are too high and that this also differs significantly from group 1 in the range 1–4 kHz. By contrast, the picture is inconsistent for the results of the loudness scaling in medium categories. In group 2, loudness of 25 CU in the low frequencies is achieved at lower levels than in group 1, while loudness of 25 CU in the high frequencies is achieved only at higher levels. One possible interpretation would be that the overall loudness interrogated during CI fitting in group 2 is predominantly achieved by the low-frequency signal components, whereas the high-frequency components tend to contribute less to the overall loudness in group 2 than in group 1. Considering the frequency content of the information-carrying consonants, this finding offers a potential explanation for the difference in speech intelligibility between groups 1 and 2. The insufficient case numbers alone do not allow a robust conclusion to be drawn here. Future studies with a larger population may confirm or exclude these possibly systematic reasons for lower speech recognition.

A GLM of the type described here can be used in different ways in the context of CI provision. The first is for predicting the outcome, and the second is in postoperative quality assurance. The former is based on preoperatively measurable influencing factors that have a certain generality, and thus can be applied to patient populations of different institutions and, after appropriate adaptation, also countries. The second application presented here is initially limited in its validity to processes within a facility or possibly to a special population. Process-related deviations from the predicted result or their causes could well apply only to individual facilities. It would also be conceivable that a CI population with narrowly defined, specific characteristics (e.g., hyperacusis, tinnitus, or inadequate compliance) would highlight other explanatory factors. The GLM derived from the present data provides a means to identify systematic causes of unmet prediction and to initiate appropriate multidisciplinary interventions in CI aftercare. However, these measures were not part of this observational study, but they offer reasoned, systematic(!) approaches to improve the quality of aftercare. The exclusion of preoperatively not foreseeable limitations of outcome due to (not yet) diagnosable retrocochlear hearing disorders should be separated from pure fitting deficits through suitable measurement methods in the future. One possibility is objective auditory pathway diagnostics using electrophysiological methods [10, 18, 19].

## Practical conclusion

- The model described here is suitable for identifying individual factors in cochlear implant (CI) aftercare that reduce speech intelligibility in a selected CI population.
- The results show that direct conclusions can be drawn from postoperative audiometry with CI for the optimization of individual CI fitting.

## Declarations

For this article, the authors did not perform any studies on humans or animals. This retrospective data evaluation was positively decided by the ethics committee at the University Medical Center Greifswald (BB 173/21).

The supplement containing this article is not sponsored by industry.
